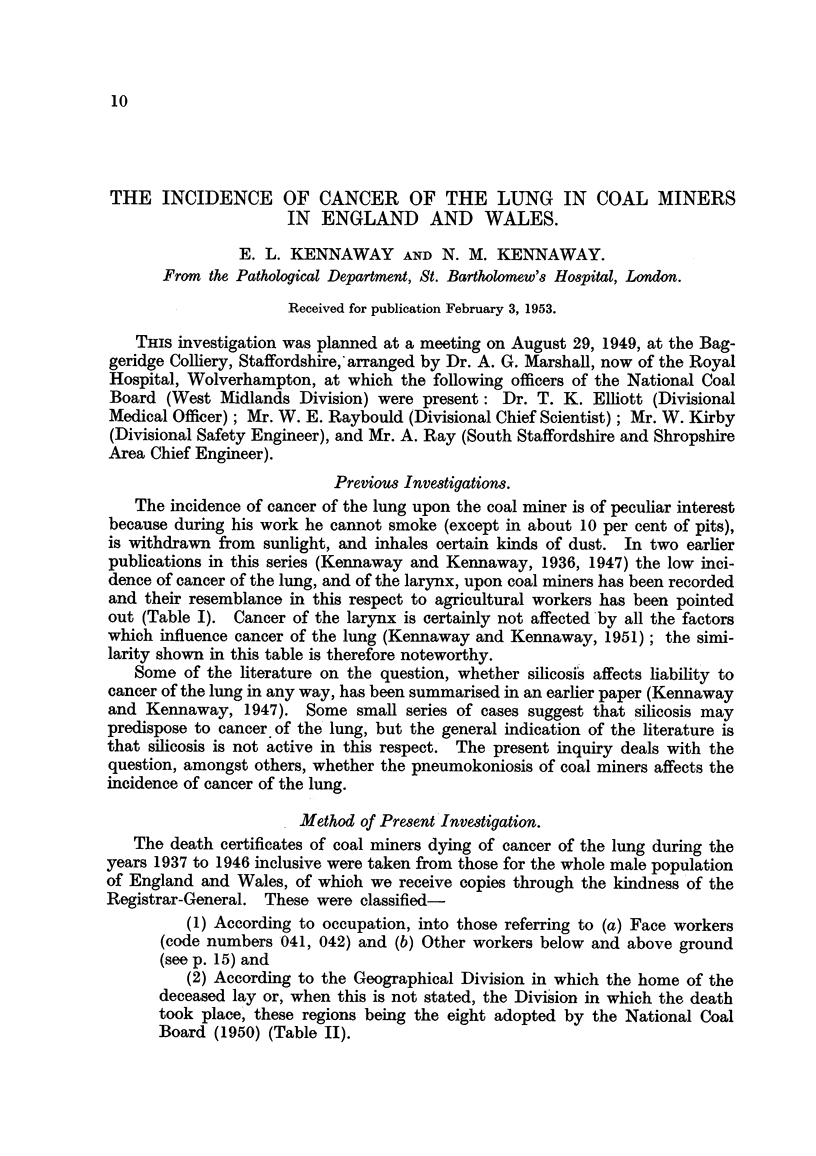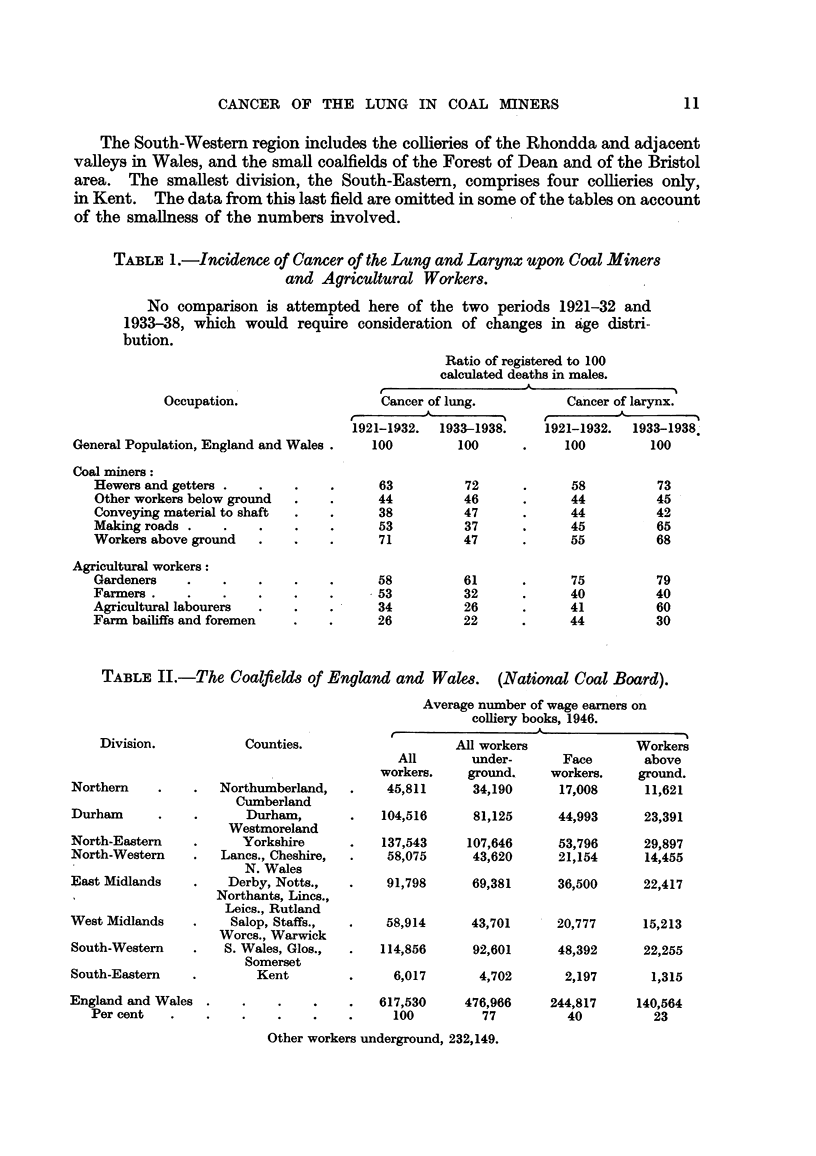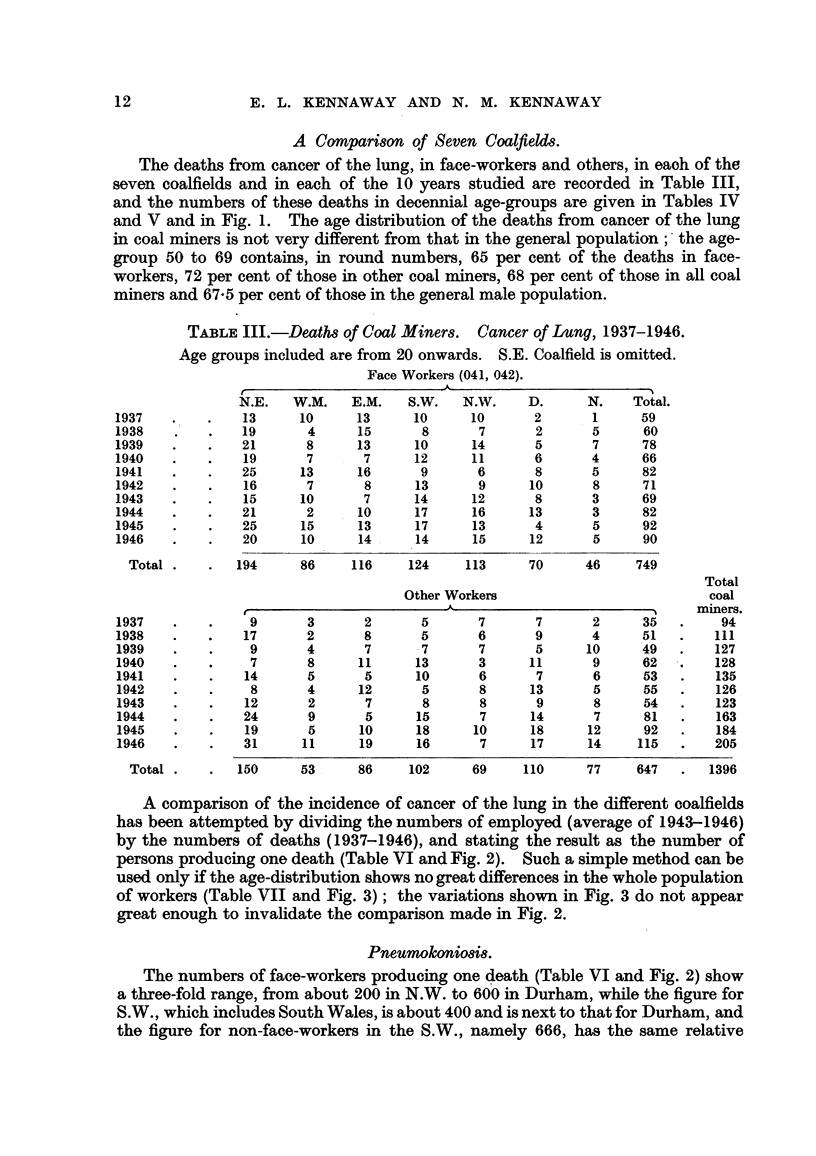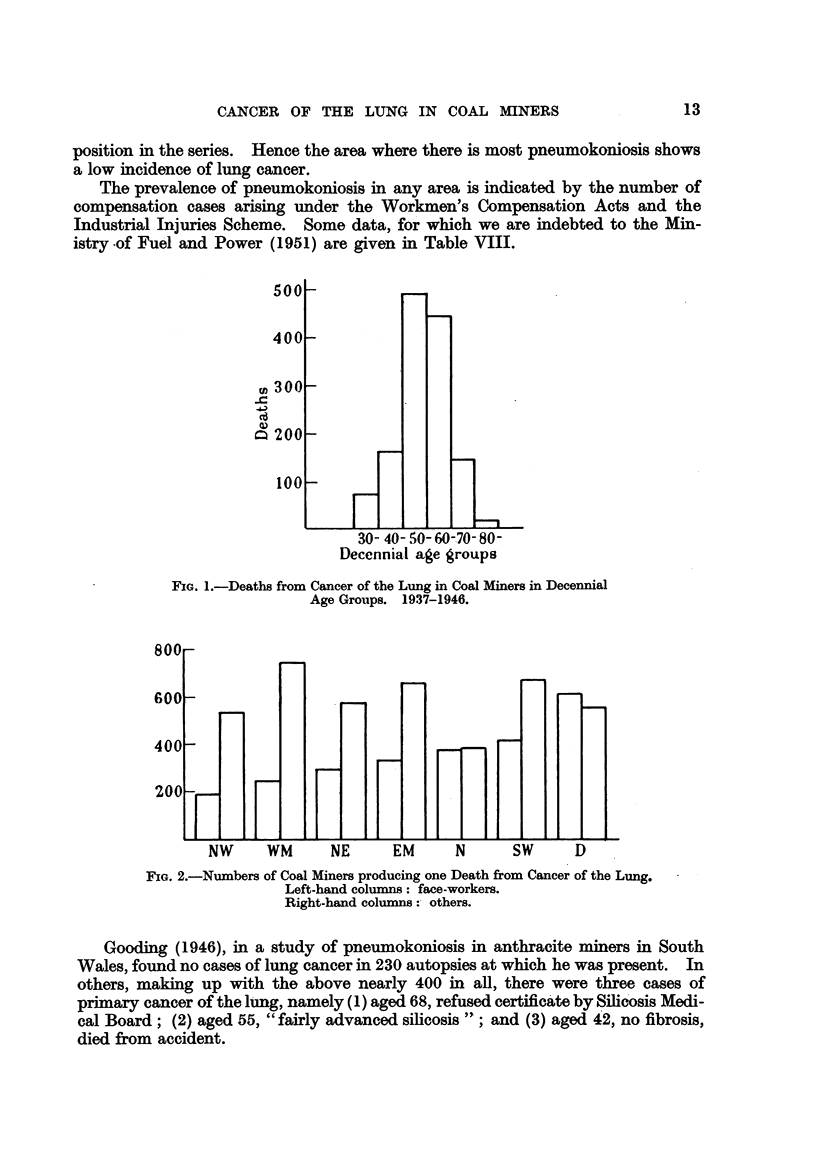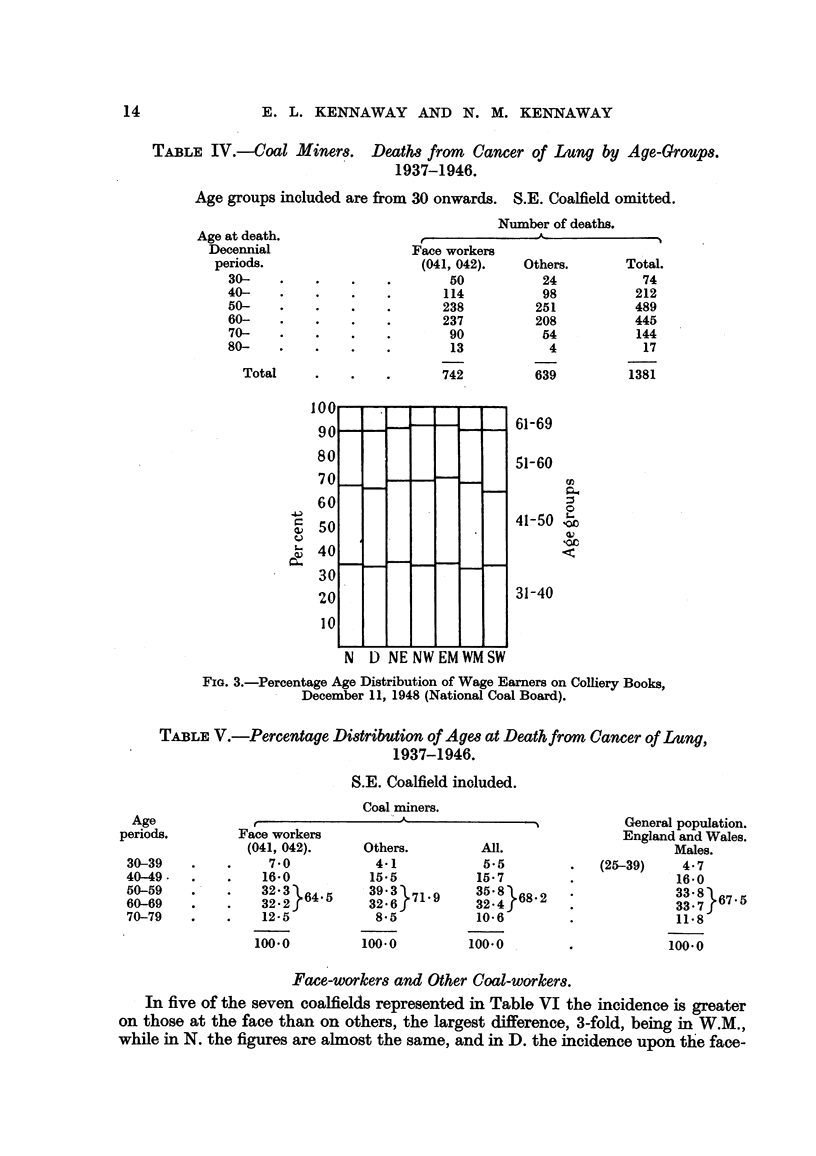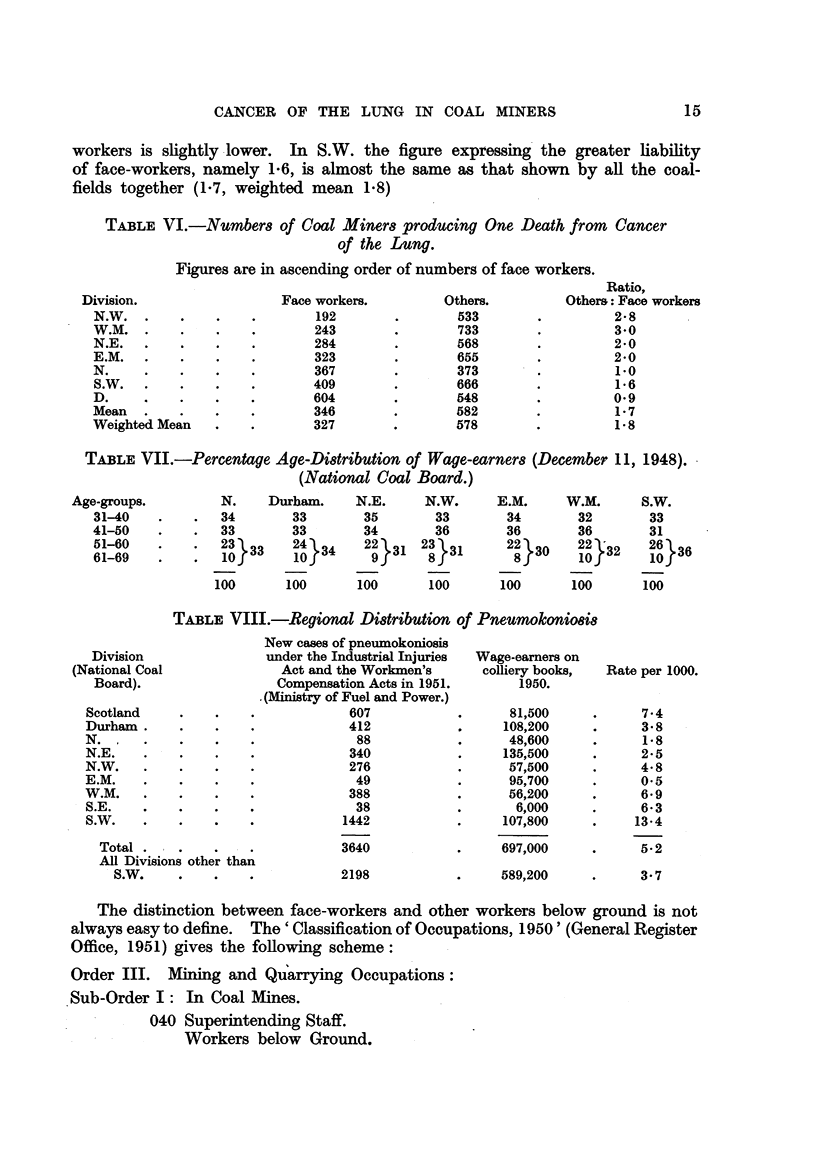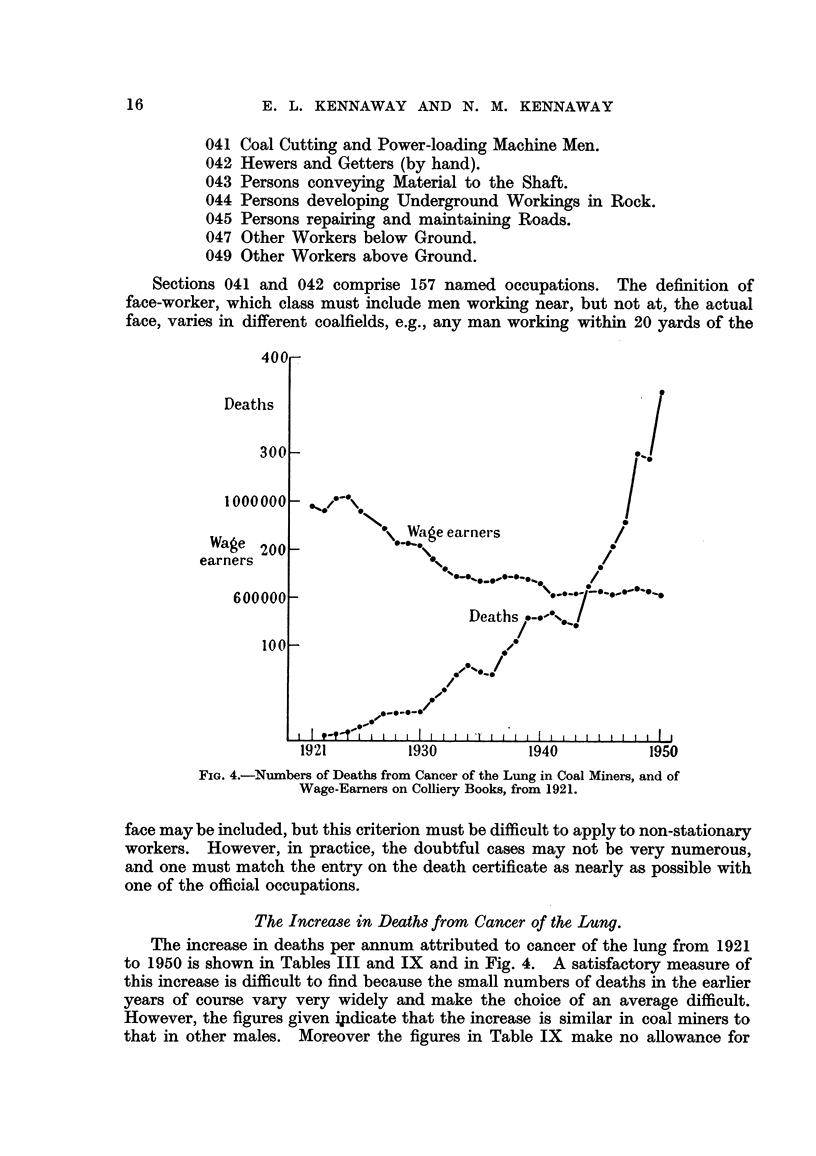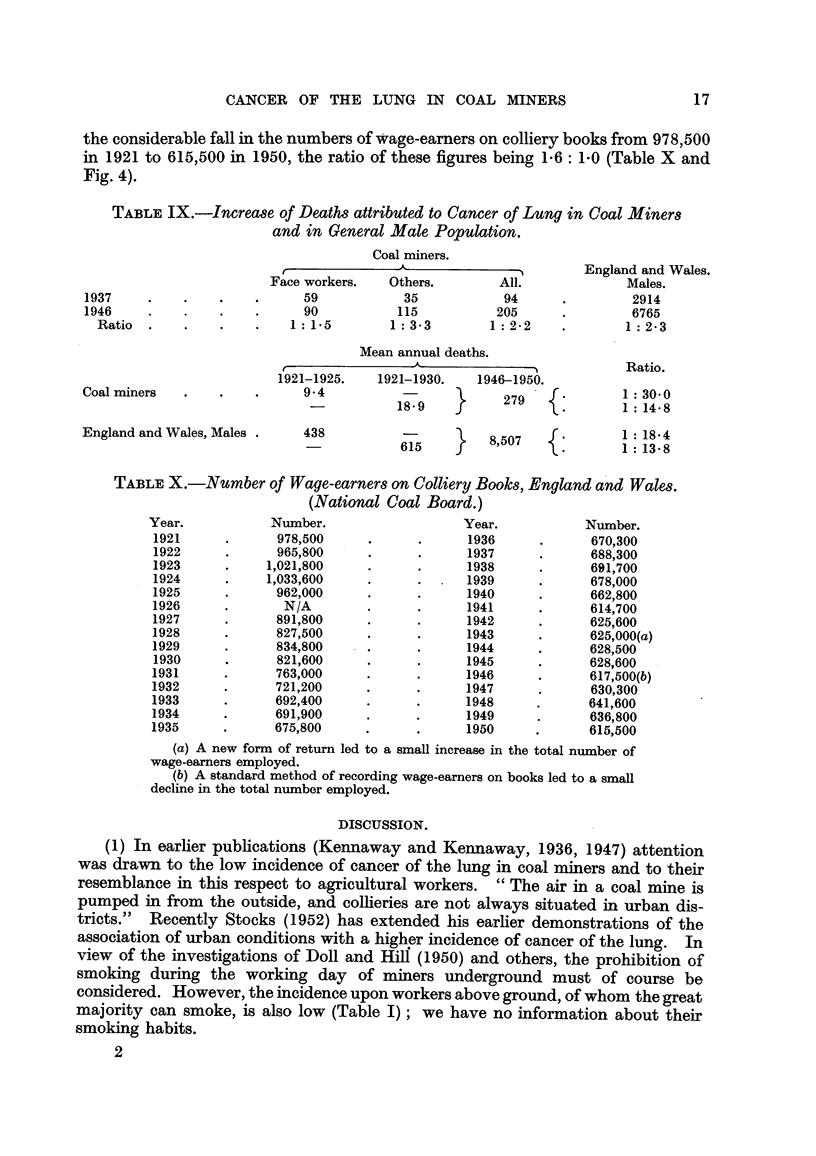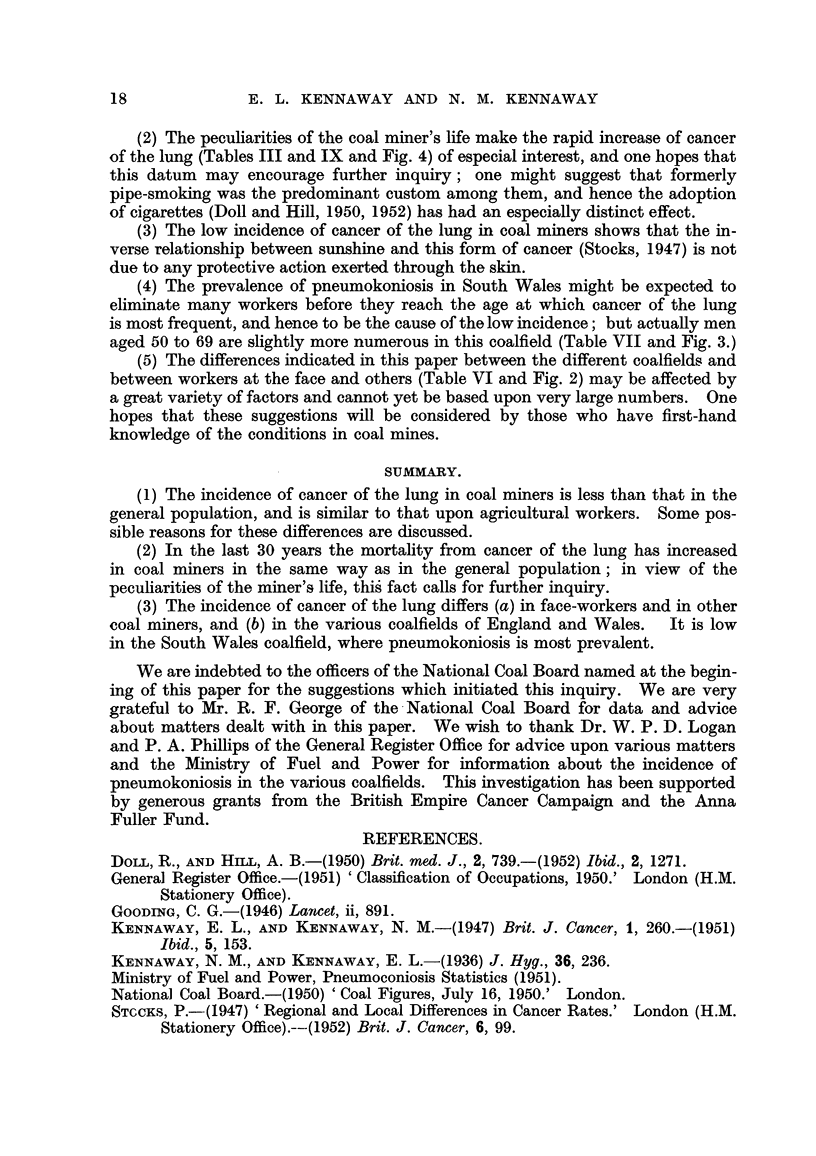# The Incidence of Cancer of the Lung in Coal Miners in England and Wales

**DOI:** 10.1038/bjc.1953.2

**Published:** 1953-03

**Authors:** E. L. Kennaway, N. M. Kennaway


### THE INCIDENCE OF CANCER OF THE LUNG IN COAL MINERS IN ENGLAND AND WALES.

E. L. KENNAWAY AND N. M. KENNAWAY.

*From the Pathological Department, St. Bartholomew's Hospital, London.*

Received for publication February 3, 1953.

THIS investigation was planned at a meeting on August 29, 1949, at the Baggeridge Colliery, Staffordshire, arranged by Dr. A. G. Marshall, now of the Royal Hospital, Wolverhampton, at which the following officers of the National Coal Board (West Midlands Division) were present: Dr. T. K. Elliott (Divisional Medical Officer); Mr. W. E. Raybould (Divisional Chief Scientist); Mr. W. Kirby (Divisional Safety Engineer), and Mr. A. Ray (South Staffordshire and Shropshire Area Chief Engineer).

### *Previous Investigations.*

The incidence of cancer of the lung upon the coal miner is of peculiar interest because during his work he cannot smoke (except in about 10 per cent of pits), is withdrawn from sunlight, and inhales certain kinds of dust. In two earlier publications in this series (Kennaway and Kennaway, 1936, 1947) the low incidence of cancer of the lung, and of the larynx, upon coal miners has been recorded and their resemblance in this respect to agricultural workers has been pointed out (Table I). Cancer of the larynx is certainly not affected by all the factors which influence cancer of the lung (Kennaway and Kennaway, 1951); the similarity shown in this table is therefore noteworthy.

Some of the literature on the question, whether silicosis affects liability to cancer of the lung in any way, has been summarised in an earlier paper (Kennaway and Kennaway, 1947). Some small series of cases suggest that silicosis may predispose to cancer of the lung, but the general indication of the literature is that silicosis is not active in this respect. The present inquiry deals with the question, amongst others, whether the pneumokoniosis of coal miners affects the incidence of cancer of the lung.

### *Method of Present Investigation.*

The death certificates of coal miners dying of cancer of the lung during the years 1937 to 1946 inclusive were taken from those for the whole male population of England and Wales, of which we receive copies through the kindness of the Registrar-General. These were classified—

(1) According to occupation, into those referring to (*a*) Face workers (code numbers 041, 042) and (*b*) Other workers below and above ground (see p. 15) and

(2) According to the Geographical Division in which the home of the deceased lay or, when this is not stated, the Division in which the death took place, these regions being the eight adopted by the National Coal Board (1950) (Table II).

The South-Western region includes the collieries of the Rhondda and adjacent valleys in Wales, and the small coalfields of the Forest of Dean and of the Bristol area. The smallest division, the South-Eastern, comprises four collieries only, in Kent. The data from this last field are omitted in some of the tables on account of the smallness of the numbers involved.

TABLE I.—*Incidence of Cancer of the Lung and Larynx upon Coal Miners and Agricultural Workers.*

No comparison is attempted here of the two periods 1921–32 and 1933–38, which would require consideration of changes in age distribution.

| Occupation. | Ratio of registered to 100 calculated deaths in males. Cancer of lung. 1921–1932. | Cancer of lung. 1933–1938. | Cancer of larynx. 1921–1932. | Cancer of larynx. 1933–1938. |
|---|---|---|---|---|
| General Population, England and Wales | 100 | 100 | 100 | 100 |
| Coal miners: | | | | |
| Hewers and getters | 63 | 72 | 58 | 73 |
| Other workers below ground | 44 | 46 | 44 | 45 |
| Conveying material to shaft | 38 | 47 | 44 | 42 |
| Making roads | 53 | 37 | 45 | 65 |
| Workers above ground | 71 | 47 | 55 | 68 |
| Agricultural workers: | | | | |
| Gardeners | 58 | 61 | 75 | 79 |
| Farmers | 53 | 32 | 40 | 40 |
| Agricultural labourers | 34 | 26 | 41 | 60 |
| Farm bailiffs and foremen | 26 | 22 | 44 | 30 |

TABLE II.—*The Coalfields of England and Wales. (National Coal Board).*

| Division. | Counties. | Average number of wage earners on colliery books, 1946. All workers. | All workers under-ground. | Face workers. | Workers above ground. |
|---|---|---|---|---|---|
| Northern | Northumberland, Cumberland | 45,811 | 34,190 | 17,008 | 11,621 |
| Durham | Durham, Westmoreland | 104,516 | 81,125 | 44,993 | 23,391 |
| North-Eastern | Yorkshire | 137,543 | 107,646 | 53,796 | 29,897 |
| North-Western | Lancs., Cheshire, N. Wales | 58,075 | 43,620 | 21,154 | 14,455 |
| East Midlands | Derby, Notts., Northants, Lincs., Leics., Rutland | 91,798 | 69,381 | 36,500 | 22,417 |
| West Midlands | Salop, Staffs., Worcs., Warwick | 58,914 | 43,701 | 20,777 | 15,213 |
| South-Western | S. Wales, Glos., Somerset | 114,856 | 92,601 | 48,392 | 22,255 |
| South-Eastern | Kent | 6,017 | 4,702 | 2,197 | 1,315 |
| England and Wales | | 617,530 | 476,966 | 244,817 | 140,564 |
| Per cent | | 100 | 77 | 40 | 23 |

Other workers underground, 232,149.

### *A Comparison of Seven Coalfields.*

The deaths from cancer of the lung, in face-workers and others, in each of the seven coalfields and in each of the 10 years studied are recorded in Table III, and the numbers of these deaths in decennial age-groups are given in Tables IV and V and in Fig. 1. The age distribution of the deaths from cancer of the lung in coal miners is not very different from that in the general population ; the age-group 50 to 69 contains, in round numbers, 65 per cent of the deaths in face-workers, 72 per cent of those in other coal miners, 68 per cent of those in all coal miners and 67·5 per cent of those in the general male population.

TABLE III.—*Deaths of Coal Miners. Cancer of Lung*, 1937–1946.

Age groups included are from 20 onwards. S.E. Coalfield is omitted.

| | N.E. | W.M. | E.M. | S.W. | N.W. | D. | N. | Total. | Total coal miners. |
|---|---|---|---|---|---|---|---|---|---|
| | Face Workers (041, 042). | | | | | | | | |
| 1937 | 13 | 10 | 13 | 10 | 10 | 2 | 1 | 59 | |
| 1938 | 19 | 4 | 15 | 8 | 7 | 2 | 5 | 60 | |
| 1939 | 21 | 8 | 13 | 10 | 14 | 5 | 7 | 78 | |
| 1940 | 19 | 7 | 7 | 12 | 11 | 6 | 4 | 66 | |
| 1941 | 25 | 13 | 16 | 9 | 6 | 8 | 5 | 82 | |
| 1942 | 16 | 7 | 8 | 13 | 9 | 10 | 8 | 71 | |
| 1943 | 15 | 10 | 7 | 14 | 12 | 8 | 3 | 69 | |
| 1944 | 21 | 2 | 10 | 17 | 16 | 13 | 3 | 82 | |
| 1945 | 25 | 15 | 13 | 17 | 13 | 4 | 5 | 92 | |
| 1946 | 20 | 10 | 14 | 14 | 15 | 12 | 5 | 90 | |
| Total | 194 | 86 | 116 | 124 | 113 | 70 | 46 | 749 | |
| | Other Workers | | | | | | | | |
| 1937 | 9 | 3 | 2 | 5 | 7 | 7 | 2 | 35 | 94 |
| 1938 | 17 | 2 | 8 | 5 | 6 | 9 | 4 | 51 | 111 |
| 1939 | 9 | 4 | 7 | 7 | 7 | 5 | 10 | 49 | 127 |
| 1940 | 7 | 8 | 11 | 13 | 3 | 11 | 9 | 62 | 128 |
| 1941 | 14 | 5 | 5 | 10 | 6 | 7 | 6 | 53 | 135 |
| 1942 | 8 | 4 | 12 | 5 | 8 | 13 | 5 | 55 | 126 |
| 1943 | 12 | 2 | 7 | 8 | 8 | 9 | 8 | 54 | 123 |
| 1944 | 24 | 9 | 5 | 15 | 7 | 14 | 7 | 81 | 163 |
| 1945 | 19 | 5 | 10 | 18 | 10 | 18 | 12 | 92 | 184 |
| 1946 | 31 | 11 | 19 | 16 | 7 | 17 | 14 | 115 | 205 |
| Total | 150 | 53 | 86 | 102 | 69 | 110 | 77 | 647 | 1396 |

A comparison of the incidence of cancer of the lung in the different coalfields has been attempted by dividing the numbers of employed (average of 1943–1946) by the numbers of deaths (1937–1946), and stating the result as the number of persons producing one death (Table VI and Fig. 2). Such a simple method can be used only if the age-distribution shows no great differences in the whole population of workers (Table VII and Fig. 3) ; the variations shown in Fig. 3 do not appear great enough to invalidate the comparison made in Fig. 2.

### *Pneumokoniosis.*

The numbers of face-workers producing one death (Table VI and Fig. 2) show a three-fold range, from about 200 in N.W. to 600 in Durham, while the figure for S.W., which includes South Wales, is about 400 and is next to that for Durham, and the figure for non-face-workers in the S.W., namely 666, has the same relative position in the series. Hence the area where there is most pneumokoniosis shows a low incidence of lung cancer.

The prevalence of pneumokoniosis in any area is indicated by the number of compensation cases arising under the Workmen's Compensation Acts and the Industrial Injuries Scheme. Some data, for which we are indebted to the Ministry of Fuel and Power (1951) are given in Table VIII.

Fig. 1.—Deaths from Cancer of the Lung in Coal Miners in Decennial Age Groups. 1937–1946.

Fig. 2.—Numbers of Coal Miners producing one Death from Cancer of the Lung.
Left-hand columns : face-workers.
Right-hand columns : others.

Gooding (1946), in a study of pneumokoniosis in anthracite miners in South Wales, found no cases of lung cancer in 230 autopsies at which he was present. In others, making up with the above nearly 400 in all, there were three cases of primary cancer of the lung, namely (1) aged 68, refused certificate by Silicosis Medical Board ; (2) aged 55, "fairly advanced silicosis" ; and (3) aged 42, no fibrosis, died from accident.

TABLE IV.—*Coal Miners. Deaths from Cancer of Lung by Age-Groups.* 1937–1946.

Age groups included are from 30 onwards. S.E. Coalfield omitted.

| Age at death. Decennial periods. | Number of deaths. Face workers (041, 042). | Others. | Total. |
|---|---|---|---|
| 30– . . . . . | 50 | 24 | 74 |
| 40– . . . . . | 114 | 98 | 212 |
| 50– . . . . . | 238 | 251 | 489 |
| 60– . . . . . | 237 | 208 | 445 |
| 70– . . . . . | 90 | 54 | 144 |
| 80– . . . . . | 13 | 4 | 17 |
| Total . . . | 742 | 639 | 1381 |

FIG. 3.—Percentage Age Distribution of Wage Earners on Colliery Books, December 11, 1948 (National Coal Board).

TABLE V.—*Percentage Distribution of Ages at Death from Cancer of Lung,* 1937–1946.

S.E. Coalfield included.

| Age periods. | Coal miners. Face workers (041, 042). | | Others. | | All. | | General population. England and Wales. Males. | |
|---|---|---|---|---|---|---|---|---|
| 30–39 . . | 7·0 | | 4·1 | | 5·5 | | (25–39) 4·7 | |
| 40–49 . . | 16·0 | | 15·5 | | 15·7 | | 16·0 | |
| 50–59 . . | 32·3 | 64·5 | 39·3 | 71·9 | 35·8 | 68·2 | 33·8 | 67·5 |
| 60–69 . . | 32·2 | | 32·6 | | 32·4 | | 33·7 | |
| 70–79 . . | 12·5 | | 8·5 | | 10·6 | | 11·8 | |
| | 100·0 | | 100·0 | | 100·0 | | 100·0 | |

*Face-workers and Other Coal-workers.*

In five of the seven coalfields represented in Table VI the incidence is greater on those at the face than on others, the largest difference, 3-fold, being in W.M., while in N. the figures are almost the same, and in D. the incidence upon the faceworkers is slightly lower. In S.W. the figure expressing the greater liability of face-workers, namely 1·6, is almost the same as that shown by all the coalfields together (1·7, weighted mean 1·8)

TABLE VI.—*Numbers of Coal Miners producing One Death from Cancer of the Lung.*

Figures are in ascending order of numbers of face workers.

| Division. | Face workers. | Others. | Ratio, Others : Face workers |
|---|---|---|---|
| N.W. | 192 | 533 | 2·8 |
| W.M. | 243 | 733 | 3·0 |
| N.E. | 284 | 568 | 2·0 |
| E.M. | 323 | 655 | 2·0 |
| N. | 367 | 373 | 1·0 |
| S.W. | 409 | 666 | 1·6 |
| D. | 604 | 548 | 0·9 |
| Mean | 346 | 582 | 1·7 |
| Weighted Mean | 327 | 578 | 1·8 |

TABLE VII.—*Percentage Age-Distribution of Wage-earners (December* 11, 1948). *(National Coal Board.)*

| Age-groups. | N. | Durham. | N.E. | N.W. | E.M. | W.M. | S.W. |
|---|---|---|---|---|---|---|---|
| 31–40 | 34 | 33 | 35 | 33 | 34 | 32 | 33 |
| 41–50 | 33 | 33 | 34 | 36 | 36 | 36 | 31 |
| 51–60 | 23 }33 | 24 }34 | 22 }31 | 23 }31 | 22 }30 | 22 }32 | 26 }36 |
| 61–69 | 10 | 10 | 9 | 8 | 8 | 10 | 10 |
| | 100 | 100 | 100 | 100 | 100 | 100 | 100 |

TABLE VIII.—*Regional Distribution of Pneumokoniosis*

| Division (National Coal Board). | New cases of pneumokoniosis under the Industrial Injuries Act and the Workmen's Compensation Acts in 1951. (Ministry of Fuel and Power.) | Wage-earners on colliery books, 1950. | Rate per 1000. |
|---|---|---|---|
| Scotland | 607 | 81,500 | 7·4 |
| Durham | 412 | 108,200 | 3·8 |
| N. | 88 | 48,600 | 1·8 |
| N.E. | 340 | 135,500 | 2·5 |
| N.W. | 276 | 57,500 | 4·8 |
| E.M. | 49 | 95,700 | 0·5 |
| W.M. | 388 | 56,200 | 6·9 |
| S.E. | 38 | 6,000 | 6·3 |
| S.W. | 1442 | 107,800 | 13·4 |
| Total | 3640 | 697,000 | 5·2 |
| All Divisions other than S.W. | 2198 | 589,200 | 3·7 |

The distinction between face-workers and other workers below ground is not always easy to define. The 'Classification of Occupations, 1950' (General Register Office, 1951) gives the following scheme:

Order III. Mining and Quarrying Occupations:

Sub-Order I: In Coal Mines.

040 Superintending Staff.
Workers below Ground.

041 Coal Cutting and Power-loading Machine Men.

042 Hewers and Getters (by hand).

043 Persons conveying Material to the Shaft.

044 Persons developing Underground Workings in Rock.

045 Persons repairing and maintaining Roads.

047 Other Workers below Ground.

049 Other Workers above Ground.

Sections 041 and 042 comprise 157 named occupations. The definition of face-worker, which class must include men working near, but not at, the actual face, varies in different coalfields, e.g., any man working within 20 yards of the face may be included, but this criterion must be difficult to apply to non-stationary workers. However, in practice, the doubtful cases may not be very numerous, and one must match the entry on the death certificate as nearly as possible with one of the official occupations.

FIG. 4.—Numbers of Deaths from Cancer of the Lung in Coal Miners, and of Wage-Earners on Colliery Books, from 1921.

*The Increase in Deaths from Cancer of the Lung.*

The increase in deaths per annum attributed to cancer of the lung from 1921 to 1950 is shown in Tables III and IX and in Fig. 4. A satisfactory measure of this increase is difficult to find because the small numbers of deaths in the earlier years of course vary very widely and make the choice of an average difficult. However, the figures given indicate that the increase is similar in coal miners to that in other males. Moreover the figures in Table IX make no allowance for the considerable fall in the numbers of wage-earners on colliery books from 978,500 in 1921 to 615,500 in 1950, the ratio of these figures being 1·6 : 1·0 (Table X and Fig. 4).

TABLE IX.—*Increase of Deaths attributed to Cancer of Lung in Coal Miners and in General Male Population.*

| | Coal miners. | | | England and Wales. |
|---|---|---|---|---|
| | Face workers. | Others. | All. | Males. |
| 1937 | 59 | 35 | 94 | 2914 |
| 1946 | 90 | 115 | 205 | 6765 |
| Ratio | 1 : 1·5 | 1 : 3·3 | 1 : 2·2 | 1 : 2·3 |

| | Mean annual deaths. | | | Ratio. |
|---|---|---|---|---|
| | 1921–1925. | 1921–1930. | 1946–1950. | |
| Coal miners | 9·4 | — | 279 | 1 : 30·0 |
| | — | 18·9 | | 1 : 14·8 |
| England and Wales, Males | 438 | — | 8,507 | 1 : 18·4 |
| | — | 615 | | 1 : 13·8 |

TABLE X.—*Number of Wage-earners on Colliery Books, England and Wales. (National Coal Board.)*

| Year. | Number. | Year. | Number. |
|---|---|---|---|
| 1921 | 978,500 | 1936 | 670,300 |
| 1922 | 965,800 | 1937 | 688,300 |
| 1923 | 1,021,800 | 1938 | 691,700 |
| 1924 | 1,033,600 | 1939 | 678,000 |
| 1925 | 962,000 | 1940 | 662,800 |
| 1926 | N/A | 1941 | 614,700 |
| 1927 | 891,800 | 1942 | 625,600 |
| 1928 | 827,500 | 1943 | 625,000(*a*) |
| 1929 | 834,800 | 1944 | 628,500 |
| 1930 | 821,600 | 1945 | 628,600 |
| 1931 | 763,000 | 1946 | 617,500(*b*) |
| 1932 | 721,200 | 1947 | 630,300 |
| 1933 | 692,400 | 1948 | 641,600 |
| 1934 | 691,900 | 1949 | 636,800 |
| 1935 | 675,800 | 1950 | 615,500 |

(*a*) A new form of return led to a small increase in the total number of wage-earners employed.
(*b*) A standard method of recording wage-earners on books led to a small decline in the total number employed.

### DISCUSSION.

(1) In earlier publications (Kennaway and Kennaway, 1936, 1947) attention was drawn to the low incidence of cancer of the lung in coal miners and to their resemblance in this respect to agricultural workers. "The air in a coal mine is pumped in from the outside, and collieries are not always situated in urban districts." Recently Stocks (1952) has extended his earlier demonstrations of the association of urban conditions with a higher incidence of cancer of the lung. In view of the investigations of Doll and Hill (1950) and others, the prohibition of smoking during the working day of miners underground must of course be considered. However, the incidence upon workers above ground, of whom the great majority can smoke, is also low (Table I); we have no information about their smoking habits.

(2) The peculiarities of the coal miner's life make the rapid increase of cancer of the lung (Tables III and IX and Fig. 4) of especial interest, and one hopes that this datum may encourage further inquiry ; one might suggest that formerly pipe-smoking was the predominant custom among them, and hence the adoption of cigarettes (Doll and Hill, 1950, 1952) has had an especially distinct effect.

(3) The low incidence of cancer of the lung in coal miners shows that the inverse relationship between sunshine and this form of cancer (Stocks, 1947) is not due to any protective action exerted through the skin.

(4) The prevalence of pneumokoniosis in South Wales might be expected to eliminate many workers before they reach the age at which cancer of the lung is most frequent, and hence to be the cause of the low incidence ; but actually men aged 50 to 69 are slightly more numerous in this coalfield (Table VII and Fig. 3.)

(5) The differences indicated in this paper between the different coalfields and between workers at the face and others (Table VI and Fig. 2) may be affected by a great variety of factors and cannot yet be based upon very large numbers. One hopes that these suggestions will be considered by those who have first-hand knowledge of the conditions in coal mines.

### SUMMARY.

(1) The incidence of cancer of the lung in coal miners is less than that in the general population, and is similar to that upon agricultural workers. Some possible reasons for these differences are discussed.

(2) In the last 30 years the mortality from cancer of the lung has increased in coal miners in the same way as in the general population ; in view of the peculiarities of the miner's life, this fact calls for further inquiry.

(3) The incidence of cancer of the lung differs (*a*) in face-workers and in other coal miners, and (*b*) in the various coalfields of England and Wales. It is low in the South Wales coalfield, where pneumokoniosis is most prevalent.

We are indebted to the officers of the National Coal Board named at the beginning of this paper for the suggestions which initiated this inquiry. We are very grateful to Mr. R. F. George of the National Coal Board for data and advice about matters dealt with in this paper. We wish to thank Dr. W. P. D. Logan and P. A. Phillips of the General Register Office for advice upon various matters and the Ministry of Fuel and Power for information about the incidence of pneumokoniosis in the various coalfields. This investigation has been supported by generous grants from the British Empire Cancer Campaign and the Anna Fuller Fund.

### REFERENCES.

DOLL, R., AND HILL, A. B.—(1950) *Brit. med. J.*, **2**, 739.—(1952) *Ibid.*, **2**, 1271.

General Register Office.—(1951) ' Classification of Occupations, 1950.' London (H.M. Stationery Office).

GOODING, C. G.—(1946) *Lancet*, ii, 891.

KENNAWAY, E. L., AND KENNAWAY, N. M.—(1947) *Brit. J. Cancer*, **1**, 260.—(1951) *Ibid.*, **5**, 153.

KENNAWAY, N. M., AND KENNAWAY, E. L.—(1936) *J. Hyg.*, **36**, 236.

Ministry of Fuel and Power, Pneumoconiosis Statistics (1951).

National Coal Board.—(1950) ' Coal Figures, July 16, 1950.' London.

STOCKS, P.—(1947) ' Regional and Local Differences in Cancer Rates.' London (H.M. Stationery Office).—(1952) *Brit. J. Cancer*, **6**, 99.